# Impact of acute anorexia nervosa and of short-/long-term recovery after refeeding on the hormonal profiles of activin A, GDF-15 and follistatins

**DOI:** 10.1038/s41380-025-03245-0

**Published:** 2025-09-18

**Authors:** Stefan Ehrlich, Louisa Licht, Theresa Kolb, Carlotta Hoffmann, Evelina Stender, Friederike I. Tam, David Poitz, Veit Roessner, Stefan R. Bornstein, Nikolaos Perakakis

**Affiliations:** 1 Division of Psychological and Social Medicine and Developmental Neurosciences, Faculty of Medicine, Technische Universität Dresden, German Center for Child and Adolescent Health (DZKJ), partner site Leipzig/Dresden, Dresden, Germany; 2 Eating Disorder Treatment and Research Center, Department of Child and Adolescent Psychiatry, Faculty of Medicine, Technische Universität Dresden, Dresden, Germany; 3 Department of Internal Medicine III, University Hospital Carl Gustav Carus, Technische Universität Dresden, Dresden, Germany; 4 Institute of Clinical Chemistry and Laboratory Medicine, University Hospital and Faculty of Medicine, Technische Universität Dresden, Dresden, Germany; 5 Department of Child and Adolescent Psychiatry, Faculty of Medicine, Technische Universität Dresden, Dresden, Germany; 6 Paul Langerhans Institute Dresden (PLID), Helmholtz Center Munich, University Hospital and Faculty of Medicine, TU Dresden, Dresden, Germany; 7 German Center for Diabetes Research (DZD e.V.), Neuherberg, Germany

**Keywords:** Diagnostic markers, Psychiatric disorders, Neuroscience, Psychology

## Abstract

Anorexia Nervosa (AN) is a severe eating disorder characterized by endocrine and metabolic abnormalities. In this study, we evaluated how the concentrations of proteins recently linked with energy homeostasis might be altered in acute AN (acAN), whether their levels are associated with reproductive hormones and whether they are restored after weight recovery. Our results show that activin A, follistatin, LH and estradiol concentrations are decreased while growth/differentiation factor-15 (GDF-15) concentrations are increased in 79 females with acAN before weight restoration (acAN_T1) compared to 79 healthy control females not receiving oral contraception (HC_OCP^−^). The concentrations of all hormones were partially or completely restored after weight restoration by short-term refeeding (acAN_T2) and in 35 females after long-term ( >6 months) recovery from AN not receiving OCPs (recAN_OCP^−^). Low activin A and high GDF-15 concentrations, as in acAN_T1, were also observed in 45 healthy control females under OCP (HC_OCP^+^) compared to HC_OCP^−^. Follistatin levels were ~3-fold higher in HC_OCP^+^ and recAN_OCP^+^ (45 female recAN under OCP) compared to HC_OCP^−^ or recAN_OCP^−^ respectively. LH, FSH and estradiol concentrations were positively associated with activin A and negatively with GDF-15 and follistatin. In conclusion, we report profound alterations in GDF-15, activin A and follistatin concentrations in acAN, which are associated with the concentrations of reproductive hormones and they are regulated by OCP and weight recovery by refeeding. Our findings support the evaluation of strategies targeting these hormones (e.g. GDF-15 inhibition) in AN to potentially increase body weight and thereby facilitate the resumption of reproductive function.

## Introduction

Anorexia nervosa (AN) is a metabo-psychiatric disorder affecting up to 4% of females and up to 0.3% of males during their lifetime [1, 2]. It is a condition of chronic energy deficiency due to food restriction and/or compensatory methods resulting in underweight. Patients with AN demonstrate robust alterations in their metabolism and often develop endocrine or metabolic abnormalities [3, 4]. One of the most common endocrine adaptation observed in females with AN is the insufficiency of hypothalamic-pituitary-gonadal axis (HPG) leading to amenorrhea and infertility [5]. This insufficiency, which may be protective by conserving energy for survival rather than reproduction, is characterized by loss of LH pulsatility, resulting in hypoestrogenism [6]. Refeeding is considered the cornerstone of treatment of AN and restoration of body weight by refeeding can often also restore menstrual cycle [5, 6]. However, a significant proportion of females with AN demonstrate a chronic disease course or several relapses after weight restoration, which demand repeated refeeding treatments and can affect reproductive health in the long-term [7, 8]. In line with this, findings from previous studies suggest that reproductive health outcomes, such as pregnancy and childbirth rates are still compromised in females with AN even after weight recovery [9, 10]. Thus, it is important to detect the endocrine changes occurring during energy deficiency, which are potentially relevant for reproductive health, as well as to understand how short- or long-term weight restoration by refeeding may affect them. Of note, the inhibition of HPG axis in AN can be somewhat similar to the one achieved by oral contraception (OCP) intake. OCP inhibits ovulation through a negative feedback loop, which results in decreased gonadotropin-releasing hormone and consequently lower LH and FSH secretion. Therefore, studying woman taking OCP can serve as a complementary model for assessing the relationship of hormones with the HPG axis.

Several previous studies have focused on identifying hormonal mediators that are regulated by energy state and may have an impact on both energy homeostasis and reproductive function [5]. Among these, leptin has been more strongly associated with metabolic and reproductive health in relation to energy state [11]. We have previously demonstrated that in hypoleptinemic females with normal weight but secondary amenorrhea due to strenuous exercise and low body fat mass (a condition termed as relative energy deficiency in sports, RED-S) leptin treatment can potentially restore menstruation [12–14]. However, in these studies, leptin treatment did not restore menstruation in all females (40% non-responders) suggesting that other hormonal systems may additionally regulate reproductive function in energy deficiency independently of leptin.

Activin A belongs to the transforming growth factor β (TGF-β) superfamily and it received its name from its capacity to stimulate follicle-stimulating hormone (FSH) release from the pituitary gland [15]. Further studies identified multiple additional paracrine and autocrine effects of activin A in the pituitary gland, ovary and uterus thus revealing a complex role for activin A in the regulation of the reproductive system [16].

Follistatin and follistatin-like 3 (FSTL3) are extracellular proteins that bind activin A and abolish its functions [16, 17]. We have previously investigated the profiles of these hormones in different metabolic states [18–21]. Specifically, we showed that follistatin and FSTL3 levels are positively associated with body mass index and fat accumulation in healthy individuals [19]. Moreover, follistatin levels are reduced shortly after bariatric surgery in individuals with excessive obesity and the magnitude of this acute reduction is associated with the level of improvement of insulin sensitivity in these patients in the long-term [18]. Interestingly, we have previously observed some unexpected changes in energy deficiency states compared to obesity. Specifically, three days of complete fasting in normal weight female individuals resulted in profound decreases of activin A and FSTL3 levels, but in unexpected increase of follistatin levels [20, 22]. Similarly, normal weight females with hypothalamic amenorrhea due to RED-S had lower activin A and FSTL3 levels and higher follistatin levels compared to healthy BMI-matched controls [20]. Importantly, these hormonal alterations did not change after leptin administration indicating the presence of a second leptin-independent hormonal system, which is potentially related to reproductive function based on energy state [20, 22].

Growth/differentiation factor 15 (GDF-15) is another protein belonging to the TGF-β family that was initially associated with inflammation and immune system response [23, 24]. Recent studies have suggested that GDF-15 can regulate body weight by reducing appetite, increasing nausea and emesis and by stimulating energy expenditure [23–27]. Paradoxically, the GDF-15 levels do not decrease but rather increase during acute fasting or in RED-S, implying a different role for GDF-15 than body weight regulation in energy deficiency states [28]. Notably, GDF-15 is strongly secreted by the placenta during pregnancy and it may also affect gonadal function, but few research studies so far have evaluated the effects of GDF-15 on reproductive health [23, 24] and none of them in relation to individual energy states.

Altogether, evidence from previous studies indicate that activin A, follistatins and GDF-15 might be involved in both the regulation of energy homeostasis and reproductive function. The first aim of our study was to assess whether the concentrations of the hormones activin A, follistatin, FSTL3 and GDF-15 are altered in acutely underweight AN compared to healthy controls and whether these alterations are related to reproductive health and in particular to hormones of the pituitary-gonadal axis (LH, FSH, estradiol) and the menstrual cycle. The second aim of our study was to evaluate how short- and long-term weight restoration in AN may affect the concentrations of the above hormones and recovery of menstruation. Finally, the third aim of our study focused on acquiring additional evidence about the possible link of the investigated hormones with the HPG axis. For this purpose, we compared the hormonal alterations occurring in AN before and after short- or long-term recovery with the hormonal alterations observed in females under OCP (with no history of AN or with long-term recovery from AN).

## Patients and Methods

### Study population

In total, 279 participants, all females, were included in this study. We had specifically the following groups:

- Acute AN (**acAN**, n = 79): This group consisted of 79 participants with acAN who were admitted at child and adolescent psychiatry and psychosomatic medicine departments of the University Hospital of Dresden to undergo intensive nutritional treatment (s. Supplemental Appendix for details). Diagnosis of AN was established based on the expert form of the Structured Interview for Anorexia and Bulimia Nervosa (SIAB-EX) [29] adapted to meet DSM-5 criteria [30]. Inclusion criteria were a body mass index (BMI) below the 10th age percentile (if < 15.5 years old) or below 17.5 kg/m² (if > 15.5 years old). The patients were assessed at two different timepoints. First assessment was within 92 h of admission to the hospital (**acAN_T1)**. Second assessment was after short-term weight restoration **(acAN_T2)** defined as a BMI increase of at least 12%.
- Long-term recovered AN (**recAN**, n = 80): This group included 80 participants, 35 who were not receiving oral contraception **(recAN_OCP**^−^**)** and 45 who were receiving oral contraception **(recAN_OCP**^**+**^**)**. All participants in this group had maintained a normal BMI, defined as > 10^th^ age percentile if younger than 18 years old or above 18.5 kg/m^2^ if older than 18 years, for at least 6 months. They also reported normal menstruation, no restrictive eating habits, and no episodes of binge eating or purging for at least 6 months before the recruitment in the study.
- Healthy controls (**HC**, n = 120): This group included 120 participants, 79 who were not receiving oral contraception **(HC_OCP**^−^**)** and 41 who were receiving oral contraception **(HC_OCP**^**+**^**)**. These participants were recruited through advertisement among middle/high school and college students. Inclusion criteria included normal weight, eumenorrhea and no history of mental illness.

A schematic of the groups/study design is provided in the Supplemental Appendix (Figure S1). Exclusion criteria are described in more detail in the Supplemental Appendix and information was obtained by using the SIAB-EX [29] supplemented by our own semi-structured interview and medical records as well as the Mini International Neuropsychiatric Interview (MINI) for HC and recAN partiipants [31]. Comorbid diagnoses for patients were obtained from medical records and confirmed by an expert clinician.

All protocols received ethical approval by the local Institutional Review Board, and all participants (and, if underage, their guardians) gave written informed consent (protocol numbers: EK 39022012, 536122015, 536122015). The study was performed in accordance with the ethical standards as laid down in the 1964 Declaration of Helsinki and its later amendments.

### Clinical measures

Eating disorder-specific psychopathology was assessed with the Eating Disorder Inventory-2 (EDI-2) [32]. Presence of depressive symptoms was evaluated with the Beck Depression Inventory Version 2 (BDI-II) [33] and general levels of psychopathology with the revised Symptom Checklist 90 (SCL-90-R) [34]. Participants in the HC and recAN group reported when their last menstruation was. BMI standard deviation scores were computed to provide an age-corrected index (BMI-SDS) [35]. Study data were managed using Research Electronic Data Capture [36].

### Biochemical measurements

Venous blood samples were collected between 7 and 9 a.m. after an overnight fast (for the acAN_T1 group within 96 h of admission to treatment). Samples were centrifuged, aliquoted and stored at −80 °C until analysis. Serum concentrations of activin A, GDF15, follistatin, and FSTL3 were measured with ELISA immunoassays (all from R&D Systems, Minnesota, USA). Assay characteristics are provided in the Supplemental Appendix. FSH (Follicle-Stimulating Hormone), LH (Luteinizing Hormone), and estradiol in blood samples were measured by the Institute for Clinical Chemistry and Laboratory Medicine TU Dresden with a COBAS 8000 system from Roche Diagnostics using electrochemiluminescence assays.

### Statistical analysis

The statistical analysis was conducted using GraphPad Prism 9 (GraphPad Software Inc., La Jolla, CA) and IBM SPSS statistics 29.0.0.0 and it is described in detail in the Supplemental Appendix. Briefly, paired t-test (for normally distributed variables) or Wilcoxon signed rank-test (for non-normally distributed variables) were used to compare acAN_T1 vs acAN_T2. Unpaired t-tests (for normally distributed variables) or Mann-Whitney test (for non-normally distributed variables) were used for all the other comparisons between groups. Nine comparisons (s. Supplemental Appendix) were performed in total and thus a p < 0.05/9 (i.e. p < 0.006) was considered significant unless otherwise stated (Bonferroni correction). To account for the potential impact of age, age and smoking, as well as age and use of antidepressants, analyses of covariance (ANCOVA) were performed for all hormones, with the not normally distributed variables being logarithmically transformed first. Post-hoc supplementary analyses using Bayesian independent samples t-test were applied to further investigate the amount of evidence for the normalization hypothesis (null hypothesis), i.e. the absence of group differences between acAN_T2 vs HC and recAN vs HC. Spearman correlation analyses were performed between all investigated hormones and BMI.

## Results

### Anthropometric characteristics and symptom levels of the study groups

Participants with acutely underweight AN (acAN_T1) had similar age as the healthy controls who were not receiving oral contraception pills (HC_OCP^−^) (Table 1 and Supplemental Table 1). Participants who had recovered for a longer time from AN and irrespectively of OCP intake (both recAN_OCP^−^ and recAN_OCP^+^), as well as HC receiving contraception pills (HC_OCP^+^) were approximately six to seven years older compared to acAN_T1 and to HC_OCP^−^. The acAN_T1 group had significantly lower BMI and BMI-SDS, as expected, compared to all other groups. As per study design, after the nutritional intervention participants had a significantly higher BMI and BMI-SDS (acAN_T2 > acAN_T1), which was nonetheless still lower compared to the BMI and BMI-SDS of the HC_OCP^−^. RecAN_OCP^−^ group had similar BMI and BMI-SDS with HC_OCP^−^. As expected, the highest EDI-2 and BDI-II scores were observed in acAN_T1 but decreased at acAN_T2. Long-term recovery (recAN_OCP^−^ group) was characterized by even lower EDI-2 and BDI-II scores, without though reaching the levels observed in HC_OCP^−^ group.

**Table 1:** Anthropometric characteristics and symptom scores of the study groups.

		acAN_T1	acAN_T2	recAN_OCP^−^	HC_OCP^−^	recAN_OCP^+^	HC_OCP^+^
Group		1	2	3	4	5	6
N		79	79	35	79	45	41
Age (years)		15.73 ± 0.22	15.97 ± 0.022	22.57 ± 0.65	16.20 ± 0.32	22.22 ± 0.48	21.72 ± 0.55
	P	2, 3, 5, 6	1	1, 4	3, 6	1	1, 4
Age onset AN		14.09 ± 0.21	14.09 ± 0.21	14.85 ± 0.48		14.53 ± 0.28	
Days of treatment		110.4 ± 2.73					
Months since recovery				53.6 ± 7.4		53.5 ± 5.5	
BMI (kg/m^2^)		14.90 ± 0.14	19.04 ± 0.13	21.14 ± 0.32	20.61 ± 0.26	21.23 ± 0.30	21.69 ± 0.37
	P	2, 3, 4, 5, 6	1, 4	1	1, 2	1	1
BMI-SDS		−2.93 ± 0.11	−0.604 ± 0.056	−0.39 ± 0.12	−0.028 ± 0.08	−3.71 ± 0.09	−0.18 ± 0.10
	P	2, 3, 4, 5, 6	1, 4	1	1, 2	1	1
EDI-2		210 ± 4.99 N = 76	197.5 ± 5.7 N = 75	175.7 ± 7.5 N = 34	139.6 ± 3.26 N = 75	158.5 ± 7.15 N = 42	130.3 ± 4.43 N = 34
	P	2, 3, 4, 5, 6	1, 4	1, 4	1, 2, 3	1	1
BDI-II		23.06 ± 1.21 N = 77	16.31 ± 1.36 N = 75	11.12 ± 1.82 N = 33	4.91 ± 0.63 N = 75	8.30 ± 1.39 N = 43	3.11 ± 0.63 N = 35
	P	2, 3, 4, 5, 6	1, 4	1, 4	1, 2, 3	1	1
Smoking Yes/no		1/78	3/76	7/28	4/75	11/34	6/35
Anti-depressants (yes/no)		0/79	3/76	6/29	0/79	1/44	0/41

Mean ± Std. Error of Mean are reported. acAN_T1, acute anorexia nervosa at timepoint 1; acAN_T2, acute anorexia nervosa at timepoint 2 (after short-term refeeding); recAN, long-term recovered anorexia nervosa; HC, healthy control group; OCP^−^, no oral contraception; OCP^+^, with oral contraception. The groups are coded from 1 to 6 and P indicates only the groups with which each respective group differs significantly at a p-value adjusted for multiple comparisons, i.e. p < 0.006. The comparisons were 9 in total: acAN_T1 (1) vs each other group [2–6], acAN_T2 [2] vs HC_OCP^−^ [4], recAN_OCP^−^ [3] vs HC_OCP^−^ [4], recAN_OCP^−^ [3] vs recAN_OCP^+^ [5], HC_OCP^−^ [4] vs HC_OCP^+^ [6]. For the exact p-values for each comparison refer to supplemental Table 1.

### Acute AN is characterized by insufficiency of the pituitary-gonadal axis and restoration of it after short- or long-term weight restoration

LH and estradiol concentrations were lower in acAN_T1 compared to HC_OCP^−^ and similar to the concentrations observed in HC_OCP^+^ (Table 2). Most of the participants in the acAN_T1 group had no menstruation in the last three months. Short-term weight gain during refeeding (acAN_T2) was characterized by an increase of LH, and estradiol levels (acAN_T2 > acAN_T1) and resumption of menstruation in some but not all patients. Females after long-term recovery from AN and menorrhea (recAN_OCP^−^) had similar levels of LH and estradiol compared to HC_OCP^−^ (Table 2 and Supplemental Table 2). The null hypothesis was verified using Bayesian statistics (Supplemental Table 3). FSH concentrations were lower in acAN_T1 compared to HC_OCP^−^ (after adjusting for age) and similar to the concentrations observed in both HC_OCP^+^ and recAN_OCP^+^ (Table 2 and Supplemental Table 2). Otherwise, results were highly similar after adjusting for age, smoking or use of antidepressants.

**Table 2:** Plasma concentrations of sex hormones and history of menstruations.

		acAN_T1	acAN_T2	recAN_OCP^−^	HC_OCP^−^	recAN_OCP^+^	HC_OCP^+^
Group		1	2	3	4	5	6
N		79	79	35	79	45	41
LH (U/L)		2.51 ± 0.47	9.28 ± 1.12	11.40 ± 2.57	9.31 ± 1.04	3.21 ± 0.52	4.07 ± 0.66
	**P**	2, 3, 4, 6	1	1, 5	1, 6	3	1, 4
	**P**^**A**^	2, 3, 4	1	1, 5	1, 6	3	4
	**P**^**AB**^	2, 3, 4	1	1, 5	1, 6	3	4
	**P**^**AC**^	2, 3, 4	1	1, 5	1, 6	3	4
FSH (U/L)		4.578 ± 0.43	5.89 ± 0.31	6.14 ± 0.48	5.62 ± 0.28	3.19 ± 0.38	3.55 ± 0.40
	**P**	ns	ns	5	6	3	4
	**P**^**A**^	2, 4	1	5	1,6	3	4
	**P**^**AB**^	2, 4	1	5	1	3	ns
	**P**^**AC**^	2, 4	1	5	1, 6	3	4
Estradiol (pmol/L)		105.7 ± 25.13	250.1 ± 35.57	424.5 ± 76.52	360.5 ± 37.63	93.33 ± 16.32	98.62 ± 15.43
	**P**	2, 3, 4	1, 4	1, 5	1, 2, 6	3	4
	**P**^**A**^	2, 4	1, 4	5	1, 2, 6	3	4
	**P**^**AB**^	2, 4	1, 4	5	1, 2, 6	3	4
	**P**^**AC**^	2, 4	1, 4	5	1, 2, 6	3	4
Leptin (ng/ml)		1.72 ± 0.30 N = 76	12.60 ± 0.77 N = 78	7.76 ± 0.61 N = 32	11.02 ± 1.05 N = 75	9.92 ± 0.96 N = 42	14.22 ± 1.55 N = 35
	**P**	2, 3, 4, 5, 6	1	1	1	1	1
	**P**^**A**^	2, 3, 4, 5, 6	1, 4	1	1,2	1	1
	**P**^**AB**^	2, 3, 4, 5, 6	1	1	1	1	1
	**P**^**AC**^	2, 3, 4, 5, 6	1	1	1	1	1
Menorrhea +	(y/n/u)	(10/56/13)	(19/48/12)	(34/0/1)	(76/0/3)	(43/0/2)	(39/0/2)
Days since last menstruation		24.63 ± 9.30 N = 8	12.63 ± 1.73 N = 16	16.29 ± 1.63 N = 34	16.59 ± 1.58 N = 74	17.52 ± 1.95 N = 42	12.39 ± 1.14 N = 38
	**P**	ns	ns	ns	ns	ns	ns
McNemar’s Test		p-value(2tail) 0.0133					

acAN_T1, acute anorexia nervosa at timepoint 1; acAN_T2, acute anorexia nervosa at timepoint 2 (after short-term refeeding); recAN, long-term recovered anorexia nervosa; HC, healthy control group; OCP^−^, no oral contraception; OCP^+^, with oral contraception. The groups are coded from 1 to 6 and P indicates only the groups with which each respective group differs significantly at a p-value adjusted for multiple comparisons, i.e. p < 0.006. The comparisons were 9 in total: acAN_T1 [1] vs each other group [2–6], acAN_T2 [2] vs HC_OCP^−^ [4], recAN_OCP^−^ [3] vs HC_OCP^−^ [4], recAN_OCP^−^ [3] vs recAN_OCP^+^ [5], HC_OCP^−^ [4] vs HC_OCP^+^ [6]. P^A^ indicates additional adjustment for age; P^AB^ indicates additional adjustments for age and current smoking, P^AC^ indicates additional adjustment for age and antidepressant use; no participants were taking antidepressants in comparisons of groups 1 vs 4, 1 vs 6 and 4 vs 6, thus P^AC^ is the same as P^A^ in these comparisons. LH, FSH, estradiol different N (T1 = 60, T2 = 60, recAN_OCP^−^ = 33, HC_OCP^−^ = 72, recAN_OCP^+^ = 44, HC_OCP^+^ = 39); OCP either progesterone, estrogen, or both. Menorrhea^+^: Self-reported menstrual cycle in the last 3 months, y = yes, n = no, u = unknown, not reported. For participants under OCP (OCP^+^), menstruation refers to withdrawal bleeding. Mean ± Std. Error of Mean are presented. Different n for Menorrhea+ vs Days since last menstruation is due to inability of few study participants to remember the exact date of last Menstruation. For the exact p-values for each comparison refer to supplemental Table 2.

### Activin A, GDF-15 and follistatin concentrations are altered in acute AN, they are (partially) restored after short- or long-term recovery and they are affected by OCPs

Activin A levels were approximately 40% lower and GDF-15 levels approximately 15% higher in acAN_T1 compared to HC_OCP^−^ (Table 3 and Supplemental Table 4). Importantly, in acAN_T1, the lowest activin A, LH and estradiol levels were observed in patients who had amenorrhea (Supplemental Table 5). Similar differences (lower activin A and higher GDF-15 concentrations) were observed under oral contraception (HC_OCP^+^ and recAN_OCP^+^ compared to HC_OCP^−^ and recAN_OCP^−^ respectively; Table 3). Partial/short-term weight restoration as observed in acAN_T2 led to a decrease of GDF-15 to the levels observed in HC_OCP^−^ (null hypothesis was verified using Bayesian statistics) and to an increase of activin A concentrations even above the HC_OCP^−^ levels (Table 3 and Supplemental Table 3). These changes in the concentrations of both hormones were independent of resumption of menses (Table 3 and Supplemental Tables 5). Follistatin levels tended to be lower in acAN_T1 compared to HC_OCP^−^ (p-value unadjusted for multiple comparisons = 0.029, Supplementary Table 4) and in acAN_T1 patients with amenorrhea compared to acAN_T1 patients with menorrhea (Supplemental Table 4). They modestly increased during short-term weight restoration (acAN_T2 concentrations ~15% higher compared to acAN_T1 concentrations) (Table 3). Importantly, follistatin concentrations were 4−5 fold elevated in participants under oral contraception (HC_OCP^+^ and recAN_OCP^+^ compared to HC_OCP^−^ and recAN_OCP^−^ respectively) (Table 3). FSTL3 concentrations were not affected by OCP or by the presence of acute AN and they increased around 15% in acAN_T2 (Table 3). No significant differences in activin A, GDF-15, follistatin and FSTL3 were detected in recAN_OCP^−^ compared to HC_OCP^−^ (Table 3 and Supplemental Table 4). Moreover, the null hypothesis was verified using Bayesian statistics (Supplemental Table 3). Otherwise, results were highly similar after adjusting for age, smoking or use of antidepressants.

**Table 3:** Plasma concentrations of activin A, GDF-15, follistatin and FSTL3.

		acAN_T1	acAN_T2	recAN_OCP^−^	HC_OCP^−^	recAN_OCP^+^	HC_OCP^+^
Group		1	2	3	4	5	6
N		79	79	35	79	45	41
Activin A (pg/mL)		148.4 ± 8.43	291.1 ± 11.73	226.2 ± 8.44	251.9 ± 12.95	168.2 ± 15.27	182.8 ± 20.02
	P	2, 3, 4	1, 4	1, 5	1, 2, 6	3	4
	P^A^	2, 3, 4, 6	1	1, 5	1	3	1
	P^AB^	2, 3, 4, 6	1	1, 5	1	3	1
	P^AC^	2, 3, 4, 6	1	1, 5	1	3	1
GDF-15 (pg/mL)		375.9 ± 15.18	319.4 ± 11.49	341.5 ± 20.94	327.8 ± 21.74	462.8 ± 32.53	483.3 ± 31.58
	P	2, 4	1	5	1, 6	3	4
	P^A^	2, 4	1	5	1, 6	3	4
	P^AB^	2, 4	1	5	1, 6	3	4
	P^AC^	2, 4	1	ns	1, 6	ns	4
Follistatin (pg/mL)		1169 ± 28.57	1352 ± 61.18	1273 ± 73.82	1500 ± 148.6	4149 ± 416.8	5279 ± 800.3
	P	2, 5, 6	1	5	6	1, 3	1, 4
	P^A^	5, 6	ns	5	6	1, 3	1, 4
	P^AB^	5, 6	ns	5	6	1, 3	1, 4
	P^AC^	5, 6	ns	5	6	1, 3	1, 4
FSTL3 (pg/mL)		4744 ± 129.0	5401 ± 142.5	5040 ± 314.4	4947 ± 138.4	4666 ± 163.1	4796 ± 188.0
	P	2	1	ns	ns	ns	ns
	P^A^	2	1	ns	ns	ns	ns
	P^AB^	2	1	ns	ns	ns	ns
	P^AC^	2	1	ns	ns	ns	ns

acAN_T1, acute anorexia nervosa at timepoint 1; acAN_T2, acute anorexia nervosa at timepoint 2 (after short-term refeeding); recAN, long-term recovered anorexia nervosa; HC, healthy control group; OCP^−^, no oral contraception; OCP^+^, with oral contraception. The groups are coded from 1 to 6 and P indicates only the groups with which each respective group differs significantly at a p-value adjusted for multiple comparisons, i.e. p < 0.006. The comparisons were 9 in total: acAN_T1 [1] vs each other group [2–6], acAN_T2 [2] vs HC_OCP^−^ [4], recAN_OCP^−^ [3] vs HC_OCP^−^ [4], recAN_OCP^−^ [3] vs recAN_OCP^+^ [5], HC_OCP^−^ [4] vs HC_OCP^+^ [6]. P^A^ indicates additional adjustment for age; P^AB^ indicates additional adjustments for age and current smoking, P^AC^ indicates additional adjustment for age and antidepressant use; no participants were taking antidepressants in comparisons of groups 1 vs 4, 1 vs 6 and 4 vs 6, thus P^AC^ is the same as P^A^ in these comparisons. For the exact p-values for each comparison refer to supplemental Table 4. Mean ± Std. Error of Mean are reported.

### Activin A, GDF-15 and follistatin concentrations are associated with sex hormones levels

A spearman correlation analysis including all the groups (but excluding the measurements of AcAN_T2) demonstrated that activin A concentrations are positively associated with the concentrations of estradiol (r = 0.533), LH (r = 0.497), and to a lesser extent with FSH (r = 0.330) (Table 4 and Supplemental Table 6). Activin A concentrations were also positively but rather modestly associated with BMI (r = 0.205) and BMI-SDS (r = 0.341). GDF-15 levels were negatively associated with estradiol (r = −0.377), LH (r = −0.347), and FSH (r = −0.355). Interestingly, GDF-15 levels were not associated with BMI or BMI-SDS. Finally, follistatin concentrations were modestly negatively associated with estradiol (r = −0.340), LH (r = −0.340), and FSH (r = −0.297), and slightly positively with BMI (r = 0.212) but not with BMI-SDS (r = 0.107). Similar associations of activin A, GDF-15 and follistatin with estradiol were observed also in a subgroup correlation analysis, including either only the healthy controls (HC_OCP^−^ and HC_OCP^+^) or only the recAN participants (recAN_OCP^+^ and recAN_OCP^−^), respectively (Supplemental Tables 7–10). Leptin itself, as expected, correlated strongly with BMI and BMI-SDS (r = 0.651, r = 0.681) and to a much lesser extent with estradiol (r = 0.251) and LH (r = 0.340), whereas among the four investigated hormones, it correlated only with Activin A (r = 0.407). FSTL3 did not correlate with any parameters, apart from activin A (r = 0.221).

**Table 4:** Correlation coefficients based on spearman correlation analyses of the plasma concentrations of activin A, GDF-15, follistatin and FSTL3 with the plasma concentrations of sex hormones, leptin and with BMI as well as BMI-SDS.

Spearman r	Activin A	GDF-15	Follistatin	FSTL3	LH	FSH	Estradiol	Leptin	BMI	BMI-SDS
Activin A	1	**−0.321**	**−0.230**	0.166	**0.490**	**0.331**	**0.574**	**0.318**	**0.306**	**0.384**
GDF-15	**−0.321**	1	**0.383**	0.098	**−0.313**	**−0.334**	**−0.401**	**−**0.020	**−**0.032	**−**0.118
Follistatin	**−0.230**	**0.383**	1	0.013	**−0.227**	**−0.328**	**−0.376**	**0.264**	**0.221**	0.147
FSTL3	0.166	0.098	0.013	1	0.119	**−**0.050	0.112	0.067	0.049	0.038
LH	**0.490**	**−0.313**	**−0.227**	0.119	1	**0.628**	**0.692**	**0.321**	**0.328**	**0.374**
FSH	**0.331**	**−0.334**	**−0.328**	**−**0.050	**0.628**	1	**0.420**	0.013	0.021	0.054
Estradiol	**0.574**	**−0.401**	**−0.376**	0.112	**0.692**	**0.420**	1	**0.226**	0.198	**0.283**
Leptin	**0.318**	**−**0.020	**0.264**	0.067	**0.321**	0.013	**0.226**	1	**0.797**	**0.789**
BMI	**0.306**	**−**0.032	**0.221**	0.049	**0.328**	0.021	0.198	**0.797**	1	**0.949**
BMI-SDS	**0.384**	**−**0.118	0.147	0.038	**0.374**	0.054	**0.283**	**0.789**	**0.949**	1

Correlations are based on all participants but excluding the data from the acAN_T2 assessments (n = 279 total; for Leptin n = 260; for LH, FSH, Estradiol n = 248). Bold font indicates significant correlations after adjusting the p-value for the number of multiple comparisons which were 28 using the Bonferroni methods (i.e. p < 1.8 × 10^−3^). The exact p-values are reported in Supplemental Table 6.

## Discussion

We demonstrate herein that activin A and follistatin concentrations are profoundly decreased while GDF-15 levels are elevated in acAN compared to HC. The concentrations of all three hormones are associated with the concentrations of the classical reproductive hormones (LH, FSH, estradiol). Consequently, they are robustly altered in participants receiving OCPs. Short-term weight gain in AN restores the concentrations of GDF-15 and follistatin to similar levels and for activin A to even higher levels than the ones observed in HC. After long-term weight recovery, no differences in activin A, GDF-15 or follistatin levels are observed compared to HC.

The observed increase in GDF-15 levels in AN is in agreement with two older studies which have also reported elevated levels of GDF-15 in smaller populations of patients with AN [37, 38]. The reported absolute concentrations in one of these studies were however three-fold higher in comparison to our study, due to the use of older assays which have been criticized for cross-reactions with other TGF-β molecules. A very recent study has also reported elevated GDF-15 levels in energy deficiency states - both during acute fasting as well as in women with chronic mild energy deprivation due to strenuous exercise [28]. Interestingly, GDF-15 has been shown to promote weight loss in mice by reducing appetite through binding to the glial cell-derived neurotrophic factor (GDNF) receptor alpha-like (GFRAL) resulting in the modulation of hindbrain function [26], as well as by increasing energy expenditure in muscle [27]. GDF-15 can further induce AN through nausea and emesis in mice [25]. Thus, the elevated GDF-15 levels in AN in humans may have detrimental effects and may contribute to or worsen the severe energy deficiency observed in the disease. On the other hand, the decrease/normalization of GDF-15 concentrations after short-term refeeding suggests that the elevated GDF-15 concentrations in AN might also serve other pathophysiological purposes beyond weight loss through appetite reduction. In the current study, we provide evidence linking GDF-15 to reproductive function in both participants with AN and healthy controls. This evidence includes negative associations of GDF-15 with LH, FSH and estradiol levels (and not with BMI), and elevated GDF-15 concentrations in participants receiving OCPs. GDF-15 has been detected in follicular fluid and is highly secreted by the placenta [23, 39–41], but its role in reproductive function and in the regulation of HPG axis remains unknown. The pharmaceutical industry is currently evaluating GDF-15 analogs for weight loss in obesity and GDF-15 inhibitors for weight gain in tumor cachexia. Our findings highlight the need to evaluate GDF-15 inhibitors as potential treatments to promote weight gain in AN. Additionally, they support the conduct of mechanistic studies to further elucidate the role of GDF-15 in reproduction and in relation to energy state.

Among the investigated hormones, activin A demonstrated the most profound reductions in acAN and specifically in participants with amenorrhea, as well as in participants receiving OCP. The role of activin A in reproduction has been extensively studied [16, 17]. Activin A is secreted by pituitary and gonads and exerts both paracrine/autocrine effects. Activin A is primarily considered a hormone that can stimulate FSH gene expression and hormone release from the pituitary gland [16, 17]. Interestingly, in our study, despite the almost 50% reduction of activin A in acAN_T1, the FSH levels were reduced only mildly compared to HC. It has been suggested that under certain conditions, activins might also affect the synthesis and release of LH [42, 43]. In agreement with this hypothesis, activin A levels were more strongly associated with LH than with FSH concentrations, which were also robustly reduced in acAN and in participants under OCP treatment. They were restored after short- or long-term weight recovery. Activin A correlated with leptin levels. However, we have previously shown that leptin administration does not change activins concentrations and thus hypoleptinemia cannot explain the low activin levels in energy deficiency [20, 22, 44]. Thus, it remains still unknown, which factors can regulate activin A synthesis and release depending on energy state.

An unexpected finding in our study are the low follistatin levels in AN. This finding is in contrast with our previous observations that follistatin levels increase during acute fasting over 72 h in normal-weight healthy females and they are elevated in normal-weight females with secondary amenorrhea due to RED-S [20, 22]. Follistatin consists of three isoforms with 288, 303, or 315 amino acids [17]. The predominant isoform in the circulation is follistatin 315 and it derives primarily from the liver [45]. Circulating follistatin is regulated acutely and positively by the glucagon-to-insulin ratio and by exercise [19, 45], which might explain the elevated follistatin levels during 72 h fasting due to acute increase of glucagon-to-insulin ratio, as well as during periods of strenuous training in females with RED-S. Follistatin has been also shown to increase insulin resistance as well as to stimulate muscle growth [17, 46]. Thus, the lower levels of follistatin in AN might serve as a beneficial compensatory mechanism contributing to insulin sensitivity and preventing energy-consuming processes such as muscle growth in severe energy-deprived states. In contrast to our observations in acAN, we observed a profound increase of follistatin concentrations in participants receiving OCPs, both in healthy ones as well as in participants who had recovered from AN. Our finding is in agreement with previous studies in females with polycystic ovary syndrome (PCOS) showing increases with different types of OCPs [47–49]. Although we do not know in our study which type of OCP the participants were receiving, the most commonly prescribed OCP is the combined estrogen-progesterone one. AcAN had similar LH, FSH, activin A and endogenous estradiol levels as healthy participants receiving OCP. Hence, the profound elevated follistatin levels in participants under OCP compared to AN or individuals who do not take OCP indicate possibly a direct modulation of follistatin levels from the external administrated estrogens-progesterone. This is plausible, given that both estrogen and progesterone receptors are expressed in the liver (primary secretion site of follistatin) and can be directly activated by OCPs [50].

Both short- and long-term weight recovery were associated with the normalization of the concentrations of activin A, GDF-15, follistatin and reproductive hormones (on the group level). However, resumption of menstruation was only achieved in a small number of participants in the short-term. This finding indicates that the restoration of certain hormones, including those investigated in our study, might be necessary but not sufficient on its own for menstrual recovery, as multiple factors contribute to this process [5]. Importantly, restoration of reproductive function requires a prolonged stable metabolic and consequently hormonal environment [51].

While the current study provides valuable insights into the relation of several hormones with markers of reproductive health in different energy states, there are several limitations to consider. We have assessed LH, FSH and estradiol at a single (albeit standardized) timepoint and thus we cannot capture changes that might occur in the pulsatile secretion profile of these hormones. Furthermore, we did not collect information about the type of OCP treatment used. The blood draw was performed in scheduled visits that were not planned according to the day of menstrual cycle. Furthermore, the current study design does not allow us to establish causality or determine the direction of the associations between metabolic hormones, sex hormones, and the menstrual cycle. Nevertheless, the current study used one of the largest study populations with acAN patients and enabled the evaluation both of short- and long-term recovery, as well as comparisons with healthy participants with both normal and OCP-inhibited pituitary-gonadal axis. The investigators who performed the hormonal measurements were blinded regarding the different groups.

In conclusion, activin A, GDF-15 and follistatin levels are altered profoundly in acAN and they are restored after short- and long-term weight recovery, following concomitant changes observed in reproductive hormones. Hence, they may contribute to the inhibition of reproductive function in states of advanced energy deficiency. Moreover, the levels of these three hormones are markedly influenced by OCP use, further underscoring their potential involvement in the hypothalamic-pituitary-gonadal axis and, consequently, in reproductive function. Notably, our study is the first to provide evidence relating GDF-15 with reproductive function both in AN and in healthy state. Currently, GDF-15 inhibitors (e.g. ponsegromab) are being evaluated in Phase II trials for the treatment of tumor cachexia, having already demonstrated evidence of efficacy (i.e. 6.6% weight gain in 12 weeks) in Phase I trials [52]. Our findings support the further evaluation of treatment strategies inhibiting GDF-15 to increase body weight and potentially restore the suppression of the HPG axis in AN. According to recent guidelines, olanzapine is the only medication with a (limited) recommendation for treating AN [53]. However, patient reluctance to initiate or adhere to olanzapine treatment, along with reports of hypo- and hyperglycemia associated with the medication, restricts its use in routine clinical practice. This underscores the need for developing new therapies that can effectively promote weight gain in AN. Additionally, after achieving weight normalization, restoring reproductive function is a crucial goal. However, drug evaluation must consider that weight restoration should precede reproductive recovery to prevent potential health complications. Finally, whether the alterations in the concentrations of these hormones contribute to the OCP-related side effects (e.g. nausea due to increased GDF-15) remains also to be assessed in future studies.

## Supplementary information

Impact of acute anorexia nervosa and of short-/long-term recovery after refeeding on the hormonal profiles of activin A, GDF-15 and follistatins

## Data Availability

The data that support the findings of this study are available from SE and NP, upon reasonable request.
