# Supplementary material for: Impact of acute anorexia nervosa and of short-/long-term recovery after refeeding on the hormonal profiles of activin A, GDF-15 and follistatins
Source: Mol Psychiatry. 2025 Sep 18;31(3):1343–50. doi: 10.1038/s41380-025-03245-0 (PMC12916494; doi:10.1038/s41380-025-03245-0)
Supplement: Supplementary file 1 — Impact of acute anorexia nervosa and of short-/long-term recovery after refeeding on the hormonal profiles of activin A, GDF-15 and follistatins [file 41380_2025_3245_MOESM1_ESM.docx]

**Supplemental Material**

1. Study description……………………………………………………………….. 1

2. Materials and Methods…………………………………………………………. 5

3. Statistical analysis………………………………………………………………. 6

4. Tables……………………………………………………………………………. 8

1. **Study description**

279 venous blood samples from volunteers participating in a longitudinal study in Dresden were included. Blood samples from all participants were collected after an overnight fasting period in-between 7 and 9 a.m., apart from the acute Anorexia Nervosa (acute AN, acAN) group, in which the blood sample was taken within 96h after admission to treatment. These participants were categorized into three distinct subgroups based on their health status.

In the first group (**acAN**), 79 participants diagnosed with AN using the expert form of the Structured Interview for Anorexia and Bulimia Nervosa (SIAB-EX) (1) which was additionally adapted to DSM-5 criteria (American Psychiatric Association 2013). Inclusion criteria for this group required a BMI below the 10^th^ age percentile (if <15.5 years old) or below 17.5 kg/m^2^ (if >15.5 years old). These 79 participants, aged 12-23 years and diagnosed with acAN, were admitted to intensive treatment at the child and adolescent psychosomatic medicine department of the University Hospital Dresden. They were recruited within 96h of admission to treatment (referred to as timepoint 1 acAN_T1) and were also reassessed after short-term weight restoration (referred to as timepoint 2 acAN_T2, with a BMI increase of at least 12%). BMI and age percentile were calculated using <https://www.pedz.de/de/rechner.html>. Weight at timepoint one (T1) was measured in the morning after an overnight fasting period on the day of the BrainArch (Tam et al., 2022).

The second group included 80 participants who had achieved long-term recovery from AN (recAN). To be eligible for this group, individuals had to maintain a normal BMI (>10^th^ age percentile if younger than 18 years or >18.5 kg/m^2^ if older than 18 years) for a minimum of 6 months. They also had to have normal menstruation, no restrictive eating habits, and no episodes of binge eating or purging for at least 6 months before the study. These participants were recruited from various locations in Germany by advertisement, word of mouth and by following up with previous patients (Wronski et al., 2022).

The third group consisted of 120 healthy controls (HC) who were eumenorrheic and mentally healthy with no history of psychiatric illness, symptoms of depression, or abnormal BMI and eating disorders. They were recruited through advertising among middle and high schools. None of the participants with acAN were taking oral contraceptives (OCP), while the recAN and HC groups were further separated based on their OCP status. The acAN and HC group were actively matched by age.


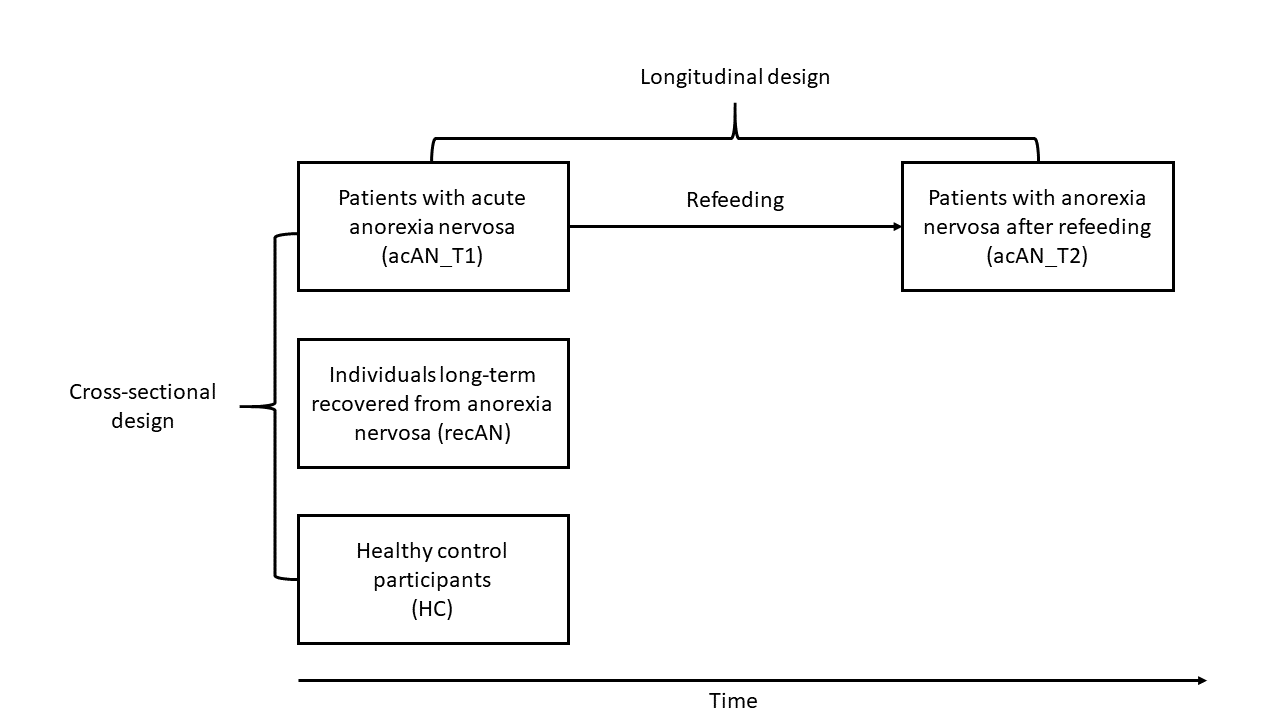


Figure S1: Study design. Cross-sectional design: Patients with acute anorexia nervosa at timepoint 1 (acAN_T1), individuals long-term recovered from anorexia nervosa (recAN), healthy control participants (HC). Longitudinal design: Follow-up examination (timepoint 2) of patients with anorexia nervosa after refeeding/short-term weight restoration (acAN_T2).

Participants in all groups were limited to females aged between 12 and 29.9 years. One of the exclusion criteria for the acAN group was significant short-term weight gain (max. 2.5 kg in 4 weeks, max. 2 kg in 2 weeks). For the recAN group individuals with obesity, indicated by a BMI exceeding the 94th age percentile or greater than 28, were not eligible for inclusion. Furthermore, participants could not be simultaneously enrolled in both the acAN and recAN cohort to prevent any overlap. Information regarding exclusion criteria was obtained from all participants using the SIAB-EX (1) supplemented by our own semi-structured interview and medical records (as well as (in HC) the German versions of the M.I.N.I. international neuropsychiatric interview or the M.I.N.I. KID interview for children and adolescents). Comorbid diagnoses in acAN were taken from medical records and confirmed by an expert clinician. All protocols received ethical approval by the local Institutional Review Board, and all participants (and, if underage, their guardians) gave written informed consent. The study was performed in accordance with the ethical standards as laid down in the 1964 Declaration of Helsinki and its later amendments.

- 1. **Acute Anorexia group (acAN) and recovery Anorexia group (recAN)**

The use of psychopharmaceuticals not permitted for this study (with some exceptions such as SSRI, SNARI, SSNRI, NASSA, and Melatonin for sleep) was also grounds for exclusion. Moreover, participants were excluded if their BMI had never been below the 10th age percentile or, for adults, BMI below 17.5 kg/m2. Importantly the presence of bulimia nervosa or binge eating disorder did not result in exclusion if these conditions occurred solely during the anorectic phase.

- 1. **Healthy group**

For the healthy group, a history of psychiatric disease at any point or the use of psychopharmaceuticals within the last 4 weeks were exclusion criteria. Extreme BMI values were also considered, leading to exclusion if a participant's BMI was exceptionally low (below the 10th age percentile or below 18.5 for adults) or high (exceeding the 94th age percentile or 28). Suspicious current weight loss, defined as more than 2.5 kg in 4 weeks or more than 2 kg in 2 weeks, was another factor leading to exclusion. Additional exclusion criteria for this group included conspicuous eating behavior, a vegan diet, or a history of bulimia nervosa, binge eating disorder, or regular binge eating.

- 1. **Additional exclusion criteria for all groups:**
- Pregnancy and Breastfeeding
- Acute inflammatory or Metabolic diseases
- Anemia
- No consent of the parents if the patient is below 18
- IQ below 85
- Not fluent in German
- A history of drug abuse
- Neurological and psychiatric conditions such as organic brain syndrome, dementia, schizophrenia, psychosis NOS, bipolar illness, or chronic medical or neurological illness that could affect appetite eating behavior or body weight
- Traumatic brain injury associated with vomiting and unconsciousness
- Malaria Prophylaxis within the last 6 weeks.
  1. **Description of the intensive inpatient refeeding**

Short-term weight restoration was achieved with completely orally intensive inpatient refeeding as described by Bargiacchi et al. (2). This included a starting daily caloric intake between 1500 and 2400 kcal with 0.5 to 2 kg per week expected body weight gain. For this purpose, a structured meal plan consisting of 3 meals, 3 snacks and a fluid intake of 2 liters per day was developed in collaboration with a nutritionist. The meal plan was adjusted for each participant based on the severity of undernutrition and on the food intake before admission to inpatient treatment. Based on the achieved weight gain, caloric intake was typically increased every 2 to 3 days in the first weeks of inpatient treatment. Hydration status was carefully monitored by regularly examining patients regarding skin tugor, edema, blood pressure and pulse. Furthermore, urine specific gravity was determined daily and electrolytes were determined every other day in the first weeks of inpatient treatment. While most patients quickly assimilated to the inpatient setting and started eating all served meals on the first or second day of inpatient treatment, a few patients refused to eat individual meals or food items. In this case, patients were encouraged to drink a corresponding quantity of a high-calorie nutrition product (Fresubin Energy Fibre Drink). All meals and snacks were supervised by the nursing staff until shortly before achieving target weight. Subsequently, the supervision was progressively reduced to encourage patients to take responsibility for adherence to the meal plan.

1. **Materials & Methods**

Plasma samples, stored at -80°C, free from prior freeze-thaw cycles, and used within one hour after the beginning of the defrosting process. Following assays were used:

- Activin A Elisa Kits from R&D Systems (Minnesota, USA), Catalog No.: DAC00B, LOT: P312341, P318823, P343412
- Follistatin Elisa Kits from R&D Systems (Minnesota, USA), Catalog No.: DFN00, LOT: P332421, P340089
- FLRG (i.e. other name for FSTL3) Elisa Kits from R&D Systems (Minnesota, USA), Catalog No.: DFLRG0, LOT: P332599, P341995
- GDF-15 Elisa Kits from R&D Systems (Minnesota, USA), Catalog No.: SGD150, LOT: P293967, P340197

The assay characteristics were:

- Follistatin intra-assay coefficient of variation (CV) 2.5%, inter-assay CV 6.8%, mean minimum detectable dose (MDD) 29 pg/ml
- Activin A intra-assay CV 4.3%, inter-assay CV 5.9%, MDD 3.67 pg/ml. It recognizes natural and recombinant human/mouse/rat Activin A
- GDF-15 intra-assay CV 2.3%, inter-assay CV 5.4%, MDD 2 pg/ml. It recognizes natural and recombinant human GDF-15
- FLRG intra-assay CV, 2.3%, inter-assay CV, 6.2%, MDD 3.68 pg/ml
- Follistatin intra-assay CV,2.5%, inter-assay CV 6.8%, MDD 29 pg/ml

No cross-reactivity or interferences in the activin A, and GDF-15 Elisa assays could be detected. Recombinant mouse follistatin cross-reacts 26% in the human follistatin assay, additionally, recombinant activin A/ activin B and activin AB interfered at concentrations ≤2 ng/mL. No significant cross-reactivity in the FLRG assay was observed, thus human activin A, activin B, activin AB, and human/mouse/rat GDF-8/Myostatin demonstrated a low level of interferences in the FLRG assay.

1. **Statistical analysis**

The statistical analysis was conducted using GraphPad Prism 9 (GraphPad Software Inc., La Jolla, CA) and IBM SPSS statistics 29.0.0.0 and it is described in detail in supplemental appendix. Briefly, the distribution for the different parameters was assessed with Shapiro-Wilk test. Values of endocrine parameters below the limit of quantification were replaced by half of the lower limit of quantification. Paired t-test (for normally distributed variables) or Wilcoxon signed rank-test (for non-normally distributed variables) were used to compare acAN_T1 vs acAN_T2. Unpaired t-tests (for normally distributed variables) or Mann-Whitney test (for non-normally distributed variables) were used for all the other comparisons between groups. 9 comparisons were performed in total and thus a p<0.05/9 (i.e. p<0.006) was considered significant unless otherwise stated (Bonferroni correction). Six groups were total. The comparisons were: acAN_T1 vs each other group, acAN_T2 vs HC_OCP^-^, recAN_OCP^-^ vs HC_OCP^-^, recAN_OCP^-^ vs recAN_OCP^+^, HC_OCP^-^ vs HC_OCP^+^. To account for the potential impact of age, an analysis of covariance (ANCOVA) was performed for all hormones. Not normally distributed variables were logarithmically transformed (natural logarithm) before performing the ANCOVA analysis.

Post-hoc supplementary analyses using Bayesian independent samples t-test were applied to further investigate the amount of evidence for the normalization hypothesis (null hypothesis), i.e. the absence of group differences between acAN_T2 vs HC_OCP^-^ and recAN_OCP^-^ vs HC_OCP^-^. Bayesian testing, particularly using the Bayes factor, offers a more nuanced understanding of statistical evidence than traditional frequentist methods. By evaluating the Bayes factor in the context of independent samples t-tests, we can not only assess the strength of evidence supporting a group effect, but also quantify the evidence for the absence of such effects or the normalization between groups, which is especially important when investigating patients after treatment, to assess the degree of recovery during this process. The strength of evidence is commonly defined according to the size of the BF and “anecdotal” (BF<3), “moderate” BF>3, and “strong” BF>10 11”. Bayesian testing was carried out using the JASP software (Version 0.18.3).

A spearman correlation analysis was performed between all investigated hormones and BMI. The study had over 80% power, two tailed and at a=0.05 to detect differences in effect sizes d of a) 0.23 (unadjusted) and 0.31 (adjusted for multiple comparisons) between acAN_T1 vs acAN_T2, b) 0.46 (unadjusted) and 0.63 (adjusted for multiple comparisons) between acAN_T1 vs HC_OCP^-^ and acAN_T2 vs HC_OCP^-^, c) 0.56 (unadjusted) and 0.76 (adjusted for multiple comparisons) between HC_OCP^-^ vs HC_OCP^+^.

1. **Tables**

**Supplemental Table 1:** Exact p-values of comparisons of anthropometric characteristics and psychopathology scores between different groups.

| **Groups** | **1=acAN_T1, 2=acAN_T2, 3=recAN_OCP^-^, 4=HC_OCP^-^, 5=recAN_OCP^+^, 6=HC_OCP^+^** | | | | | | | | | |
| --- | --- | --- | --- | --- | --- | --- | --- | --- | --- | --- |
| **Test** |  | **1 vs 2** | **1 vs 3** | **1 vs 4** | **1 vs 5** | **1 vs 6** | **2 vs 4** | **3 vs 4** | **3 vs 5** | **4 vs 6** |
| **Age (years)** | **P** | **<1x10^-10^** | **<1x10^-10^** | 0.555 | **<1x10^-10^** | **<1x10^-10^** | 0.806 | **<1x10^-10^** | 0.663 | **<1x10^-10^** |
| **BMI (kg/m^2^)** | **P** | **<1x10^-10^** | **<1x10^-10^** | **<1x10^-10^** | **<1x10^-10^** | **<1x10^-10^** | **2x10^-6^** | 0.248 | 0.838 | 0.026 |
| **BMI-SDS** | **P** | **<1x10^-10^** | **<1x10^-10^** | **<1x10^-10^** | **<1x10^-10^** | **<1x10^-10^** | **6x10^-7^** | 0.017 | 0.895 | 0.242 |
| **EDI-2** | **P** | **0.003** | **1x10^-4^** | **<1x10^-10^** | **2x10^-8^** | **<1x10^-10^** | **<1x10^-10^** | **2x10^-5^** | 0.190 | 0.069 |
| **BDI-II** | **P** | **1x10^-8^** | **2x10^-7^** | **<1x10^-10^** | **<1x10^-10^** | **<1x10^-10^** | **<1x10^-10^** | **0.002** | 0.184 | 0.050 |

acAN_T1, acute anorexia nervosa at timepoint 1; acAN_T2, acute anorexia nervosa at timepoint 2 (after short-term refeeding); recAN, long-term recovered anorexia nervosa; HC, healthy control group; OCP-, no oral contraception; OCP+, with oral contraception. The groups are coded from 1 to 6 and P indicates only the groups with which each respective group differs significantly at a p-value adjusted for multiple comparisons, i.e. p<0.006. The comparisons were 9 in total: acAN_T1 (1) vs each other group (2, 3, 4, 5, 6), acAN_T2 (2) vs HC_OCP- (4), recAN_OCP- (3) vs HC_OCP- (4), recAN_OCP- (3) vs recAN_OCP+ (5), HC_OCP- (4) vs HC_OCP+ (6).
Parametric comparisons: Age: 3 vs 5 / 5 vs 6; BMI: 1 vs 2,3,4 / 2 vs 4 / 3 vs 4; BMI-SDS: 3 vs 4,5; EDI-2: 1 vs 2,4,5 / 2 vs 4; all other comparisons were non-parametric.

**Supplementary Table 2:** Exact p-values of comparisons of the plasma concentrations of sex hormones between different groups.

| **Groups** | **1= acAN_T1, 2= acAN_T2, 3= recAN_OCP^-^, 4= HC_OCP^-^, 5= recAN_OCP^+^, 6= HC_OCP^+^** | | | | | | | | | | |
| --- | --- | --- | --- | --- | --- | --- | --- | --- | --- | --- | --- |
| **Test** |  | **1 vs 2** | **1 vs 3** | **1 vs 4** | **1 vs 5** | **1 vs 6** | **2 vs 4** | **3 vs 4** | **3 vs 5** | **4 vs 6** |  |
| **LH**  **(U/L)** | **P** | **1x10^-9^** | **<1x10^-10^** | **1x10^-10^** | 0.278 | **0.003** | 0.860 | 0.489 | **8x10^-9^** | **5x10^-7^** |  |
|  | **P^A^** | **<0.001** | **<0.001** | **<0.001** | 0.842 | 0.060 | 0.867 | 0.960 | **<0.001** | **<0.001** |  |
|  | **P^AB^** | **<0.001** | **<0.001** | **<0.001** | 0.769 | 0.015 | 0.517 | 0.386 | **<0.001** | **<0.001** |  |
|  | **P^AC^** | **<0.001** | **<0.001** | **<0.001** | 0.613 | 0.060 | 0.444 | 0.456 | **<0.001** | **<0.001** |  |
| **FSH**  **(U/L)** | **P** | 0.009 | 0.025 | 0.028 | 0.047 | 0.174 | 0.524 | 0.325 | **2x10^-5^** | **8x10^-5^** |  |
|  | **P^A^** | **0.002** | 0.185 | **0.002** | 0.028 | 0.200 | 0.565 | 0.141 | **<0.001** | **<0.001** |  |
|  | **P^AB^** | **0.002** | 0.190 | **0.001** | 0.103 | 0.651 | 0.572 | 0.077 | **<0.001** | 0.009 |  |
|  | **P^AC^** | **0.002** | 0.226 | **0.002** | 0.157 | 0.200 | 0.621 | 0.181 | **<0.001** | **<0.001** |  |
| **Estradiol**  **(pmol/L)** | **P** | **1x10^-10^** | **<1x10^-10^** | **<1x10^-10^** | 0.284 | 0.592 | **0.005** | 0.949 | **2x10^-9^** | **<1x10^-10^** |  |
|  | **P^A^** | **<0.001** | 0.018 | **<0.001** | 0.296 | 0.702 | **0.004** | 0.325 | **<0.001** | **<0.001** |  |
|  | **P^AB^** | **<0.001** | 0.018 | **<0.001** | 0.335 | 0.419 | **<0.001** | 0.998 | **<0.001** | **<0.001** |  |
|  | **P^AC^** | **<0.001** | 0.013 | **<0.001** | 0.294 | 0.702 | **0.002** | 0.829 | **<0.001** | **<0.001** |  |
| **Leptin**  **(ng/ml)** | **P** | **<1x10^-10^** | **<1x10^-10^** | **<1x10^-10^** | **<1x10^-10^** | **<1x10^-10^** | 0.011 | 0.237 | 0.200 | 0.021 |  |
|  | **P^A^** | **<0.001** | **<0.001** | **<0.001** | **<0.001** | **<0.001** | **<0.001** | 0.476 | 0.126 | 0.126 |  |
|  | **P^AB^** | **<0.001** | **<0.001** | **<0.001** | **<0.001** | **<0.001** | 0.0062 | 0.787 | 0.103 | 0.603 |  |
|  | **P^AC^** | **<0.001** | **<0.001** | **<0.001** | **<0.001** | **<0.001** | 0.010 | 0.926 | 0.185 | 0.126 |  |
| **Days since last menstruation** | **P** | 0.192 | 0.523 | 0.389 | 0.493 | 0.191 | 0.271 | 0.519 | 0.948 | 0.143 |  |

acAN_T1, acute anorexia nervosa at timepoint 1; acAN_T2, acute anorexia nervosa at timepoint 2 (after short-term refeeding); recAN, long-term recovered anorexia nervosa; HC, healthy control group; OCP^-^, no oral contraception; OCP^+^, with oral contraception. The groups are coded from 1 to 6 and P indicates only the groups with which each respective group differs significantly at a p-value adjusted for multiple comparisons, i.e. p < 0.006. The comparisons were 9 in total: acAN_T1 (1) vs each other group (2,3,4,5,6), acAN_T2 (2) vs HC_OCP^-^ (4), recAN_OCP^-^ (3) vs HC_OCP^-^ (4), recAN_OCP^-^ (3) vs recAN_OCP^+^ (5), HC_OCP^-^ (4) vs HC_OCP^+^ (6). P^A^ indicates additional adjustment for age; P^AB^ indicates additional adjustments for age and current smoking, P^AC^ indicates additional adjustment for age and antidepressant use; no participants were taking antidepressants in comparisons of groups 1 vs 4, 1 vs 6 and 4 vs 6, thus P^AC^ is the same as P^A^ in these comparisons. Each p-value refers to a comparison between two groups (indicated with group numbers). Parametric comparisons: FSH: 2 vs 4 / 3 vs 4; all other comparisons were non-parametric.

**Supplemental Table 3:** Bayesian Independent Samples T-test.

|  | **BF01** | **Error%** |
| --- | --- | --- |
| **recAN_OCP^-^ vs HC_OCP^-^** |  |  |
| LH | 3.172 | 0.019 |
| FSH | 2.956 | 0.019 |
| Estradiol | 3.322 | 0.019 |
| Activin A | 2.303 | 0.018 |
| GDF-15 | 4.381 | 0.024 |
| Follistatin | 3.033 | 0.020 |
| FSTL3 | 4.480 | 0.024 |
| **acAN_T2 vs HC_OCP^-^** |  |  |
| LH | 5.388 | 0.039 |
| FSH | 4.441 | 0.035 |
| Estradiol | 0.726 | 0.012 |
| Activin A | 0.581 | 0.011 |
| GDF-15 | 5.353 | 0.048 |
| Follistatin | 3.817 | 0.039 |
| FSTL3 | 0.536 | 0.010 |

BF01: The Bayes factor indicating the evidence for the null hypothesis of no difference between groups.

**Supplemental Table 4:** Exact p-values of comparisons of the plasma concentrations of activin A, follistatin, GDF-15, FSTL3 and FSTL1 between different groups.

| **Groups** | **1=acAN_T1, 2=acAN_T2, 3=recAN_OCP^-^, 4=HC_OCP^-^, 5=recAN_OCP^+^, 6=HC_OCP^+^** | | | | | | | | | | |
| --- | --- | --- | --- | --- | --- | --- | --- | --- | --- | --- | --- |
| **Test** |  | **1 vs 2** | **1 vs 3** | **1 vs 4** | **1 vs 5** | **1 vs 6** | **2 vs 4** | **3 vs 4** | **3 vs 5** | **4 vs 6** |  |
| **Activin A (pg/mL)** | **P** | **<1x10^-10^** | **<1x10^-10^** | **<1x10^-10^** | 0.053 | 0.037 | **0.003** | 0.385 | **1x10^-7^** | **1x10^-7^** |  |
|  | **P^A^** | **<0.001** | **<0.001** | **<0.001** | 0.550 | **0.005** | 0.016 | 0.789 | **<0.001** | 0.022 |  |
|  | **P^AB^** | **<0.001** | **<0.001** | **<0.001** | 0.613 | **0.0056** | 0.020 | 0.790 | **<0.001** | 0.029 |  |
|  | **P^AC^** | **<0.001** | **<0.001** | **<0.001** | 0.547 | **0.005** | 0.021 | 0,926 | **<0.001** | 0.022 |  |
| **GDF-15 (pg/mL)** | **P** | **2x10^-6^** | 0.226 | **6x10^-4^** | 0.025 | 0.008 | 0.711 | 0.164 | **0.004** | **1x10^-6^** |  |
|  | **P^A^** | **<0.001** | 0.050 | **0.003** | 0.573 | 0.431 | 0.802 | 0.679 | 0,0056 | **0.003** |  |
|  | **P^AB^** | **<0.001** | 0.052 | **0.001** | 0.439 | 0.517 | 0.762 | 0.725 | 0.0055 | **0.003** |  |
|  | **P^AC^** | **<0.001** | 0.046 | **0.003** | 0.572 | 0.431 | 0.976 | 0,615 | 0.007 | **0.003** |  |
| **Follistatin (pg/mL)** | **P** | **0.001** | 0.669 | 0.029 | **<1x10^-10^** | **<1x10^-10^** | 0.978 | 0.356 | **3x10^-8^** | **1x10^-8^** |  |
|  | **P^A^** | 0.022 | 0.889 | 0.011 | **<0.001** | **<0.001** | 0.557 | 0.220 | **<0.001** | **<0.001** |  |
|  | **P^AB^** | 0.032 | 0.908 | 0.011 | **<0.001** | **<0.001** | 0.692 | 0.254 | **<0.001** | **<0.001** |  |
|  | **P^AC^** | 0.011 | 0.613 | 0.011 | **<0.001** | **<0.001** | 0.570 | 0.362 | **<0.001** | **<0.001** |  |
| **FSTL3 (pg/mL)** | **P** | **1x10^-8^** | 0.603 | 0.295 | 0.922 | 0.877 | 0.024 | 0.879 | 0.463 | 0.523 |  |
|  | **P^A^** | **0.001** | 0.715 | 0.242 | 0.459 | 0.281 | 0.019 | 0.272 | 0.709 | 0.053 |  |
|  | **P^AB^** | **0.001** | 0.757 | 0.336 | 0.326 | 0.396 | 0.015 | 0.465 | 0.457 | 0.207 |  |
|  | **P^AC^** | **<0.001** | 0.622 | 0.242 | 0.360 | 0.281 | 0.013 | 0.347 | 0.594 | 0.053 |  |

acAN_T1, acute anorexia nervosa at timepoint 1; acAN_T2, acute anorexia nervosa at timepoint 2 (after short-term refeeding); recAN, long-term recovered anorexia nervosa; HC, healthy control group; OCP^-^, no oral contraception; OCP^+^, with oral contraception. The groups are coded from 1 to 6 and P indicates only the groups with which each respective group differs significantly at a p-value adjusted for multiple comparisons, i.e. p<0.006. The comparisons were 9 in total: acAN_T1 (1) vs each other group (2, 3, 4, 5, 6), acAN_T2 (2) vs HC_OCP^-^ (4), recAN_OCP^-^ (3) vs HC_OCP^-^ (4), recAN_OCP^-^ (3) vs recAN_OCP^+^ (5), HC_OCP^-^ (4) vs HC_OCP^+^ (6). P^A^ indicates additional adjustment for age; P^AB^ indicates additional adjustments for age and current smoking, P^AC^ indicates additional adjustment for age and antidepressant use; No participants were taking antidepressants in comparisons of groups 1 vs 4, 1 vs 6 and 4 vs 6, thus P^AC^ is the same as P^A^ in these comparisons. Each p-value refers to a comparison between two groups (indicated with group numbers). Parametric comparisons: FSTL3: 2 vs 4,6 / 4 vs 6; all other comparisons were non-parametric.

**Supplemental Table 5:** Comparison of the plasma concentrations of sex hormones, activin A, GDF-15, follistatin and FSTL3 in women with acAN before and after short-term weight restoration according to the presence or absence of menstruation in the last 3 months.

|  |  | acAN_T1  Menorrhea  Yes | acAN_T1  Menorrhea  No | | acAN_T2  Menorrhea  Yes | | acAN_T2  Menorrhea  No |
| --- | --- | --- | --- | --- | --- | --- | --- |
| **Group** |  | 1 | 2 | | 3 | | 4 |
| **N** |  | 10 | 56 | | 19 | | 48 |
| **Age**  **(years)** |  | 16.88 ± 0.62 | 15.94 ± 0.25 | | 16.33 ± 0.29 | | 16.23 ± 0.30 |
|  | **P** | 0.143 | | | 0.475 | | |
| **BMI**  **(Kg/m^2^)** |  | 15.43 ± 0.37 | 15.05 ± 0.15 | | 19.57 ± 0.22 | | 19.00 ± 0.154 |
|  | **P** | 0.329 | | | 0.078 | | |
| **BMI-SDS** |  | -2.86 ± 0.35 | | -2.88 ± 0.12 | -0.46 ± 0.09 | -0.67 ± 0.08 | |
|  | **P** | 0.909 | | | 0.181 | | |
| **LH**  **(U/L)** |  | 5.04 ± 1.25 | 1.90 ± 0.40 | | 12.72 ± 2.89 | | 8.05 ± 1.17 |
|  | **P** | **0.014** | | | **0.048** | | |
| **FSH**  **(U/L)** |  | 5.53 ± 1.9 | 4.60 ± 0.49 | | 5.54 ± 0.57 | | 5.93 ± 0.41 |
|  | **P** | 0.464 | | | 0.230 | | |
| **Estradiol (pmol/L)** |  | 310.9 ± 168.8 | 76.81 ± 10.88 | | 343.5 ± 84.44 | | 220.1 ± 41.57 |
|  | **P** | **0.026** | | | **0.013** | | |
| **Leptin**  **(ng/ml)** |  | 3.03 ± 1.57 | 1.46 ± 0.25 | | 16.66 ± 1.68 | | 11.46 ± 0.89 |
|  | **P** | 0.069 | | | **0.004** | | |
| **Activin A**  **(pg/mL)** |  | 182.7 ± 24.49 | 141.9 ± 10.29 | | 299.5 ± 22.27 | | 270.7 ± 14.36 |
|  | **P** | **0.019** | | | 0.287 | | |
| **GDF-15**  **(pg/mL)** |  | 372.6 ± 40.91 | 371.5 ± 14.89 | | 316.2 ± 22.26 | | 311 ± 15.98 |
|  | **P** | 0.977 | | | 0.681 | | |
| **Follistatin**  **(pg/mL)** |  | 1327 ± 84.11 | 1111 ± 32.31 | | 1275 ± 84.25 | | 1362 ± 92.47 |
|  | **P** | **0.013** | | | 0.788 | | |
| **FSTL3**  **(pg/mL)** |  | 5546 ± 502.1 | 4638 ± 140.1 | | 5602 ± 407.2 | | 5260 ± 157.0 |
|  | **P** | **0.024** | | | 0.340 | | |

Menorrhea refers to the presence of a menstrual cycle in the last 3 months. acAN_T1, acute anorexia nervosa at timepoint 1; acAN_T2, acute anorexia nervosa at timepoint 2 (after short-term refeeding); Mean ± Std. Error of Mean are reported.

Parametric comparisons (Groups): BMI= 1 vs 2; Activin A: 3 vs 4; GDF-15: 1 vs 2, Follistatin: 1 vs 2; FSTL3: 3 vs 4; leptin: 3 vs 4; all other comparisons were non-parametric.

**Supplemental Table 6:** All groups (but excluding acAN_T2 data): Exact p-values of correlation coefficients based on spearman correlation analyses of the plasma concentrations of activin A, follistatin, GDF-15, and FSTL3 with the plasma concentrations of sex hormones and with BMI.

| P-values | Activin A | GDF-15 | Follistatin | FSTL3 | LH | FSH | Estradiol | Leptin | BMI | BMI-SDS |
| --- | --- | --- | --- | --- | --- | --- | --- | --- | --- | --- |
| Activin A |  | **4x10^-8^** | **1x10^-4^** | 0.005 | **2x10^-16^** | **9x10^-8^** | **4x10^-23^** | **2x10^-7^** | **2x10^-7^** | **3x10^-11^** |
| GDF-15 | **4x10^-8^** |  | **4x10^-11^** | 0.103 | **5x10^-7^** | **7x10^-8^** | **5x10^-11^** | 0.745 | 0.590 | 0.049 |
| Follistatin | **1x10^-4^** | **4x10^-11^** |  | 0.826 | **3x10^-4^** | **1x10^-7^** | **1x10^-9^** | **2x10^-5^** | **2x10^-4^** | 0.014 |
| FSTL3 | 0.005 | 0.103 | 0.826 |  | 0.062 | 0.431 | 0.078 | 0.284 | 0.419 | 0.532 |
| LH | **2x10^-16^** | **5x10^-7^** | **3x10^-4^** | 0.062 |  | **1x10^-28^** | **1x10^-36^** | **7x10^-7^** | **1x10^-7^** | **1x10^-9^** |
| FSH | **9x10^-8^** | **7x10^-8^** | **1x10^-7^** | 0.431 | **1x10^-28^** |  | **5x10^-12^** | 0.840 | 0.742 | 0.395 |
| Estradiol | **4x10^-23^** | **5x10^-11^** | **1x10^-9^** | 0.078 | **1x10^-36^** | **5x10^-12^** |  | **6x10^-4^** | 0.002 | **6x10^-6^** |
| Leptin | **2x10^-7^** | 0.745 | **2x10^-5^** | 0.284 | **7x10^-7^** | 0.840 | **6x10^-4^** |  | **2x10^-58^** | **2x10^-56^** |
| BMI | **2x10^-7^** | 0.590 | **2x10^-4^** | 0.419 | **1x10^-7^** | 0.742 | 0.002 | **2x10^-58^** |  | **2x10^-140^** |
| BMI-SDS | **3x10^-11^** | 0.049 | 0.014 | 0.532 | **1x10^-9^** | 0.395 | **6x10^-6^** | **2x10^-56^** | **2x10^-140^** |  |

Correlations are based on all participants but excluding the data from the acAN_T2 assessments (n=279 total; for Leptin n=260; for LH, FSH, Estradiol n=248).

Bold font indicates significant correlations after adjusting the p-value for the number of multiple comparisons which were 28 (i.e. p<1.8x10^-3^).

**Supplemental Table 7:** HC group (both HC_OCP+ and HC_OCP-): Correlation coefficients based on spearman correlation analyses of the plasma concentrations of activin A, GDF-15, follistatin and FSTL3 with the plasma concentrations of sex hormones, leptin and with BMI as well as BMI-SDS.

| Spearman r | Activin A | GDF-15 | Follistatin | FSTL3 | LH | FSH | Estradiol | Leptin | BMI | BMI-SDS |
| --- | --- | --- | --- | --- | --- | --- | --- | --- | --- | --- |
| Activin A | 1 | **-0.358** | **-0.502** | 0.203 | **0.354** | **0.310** | **0.473** | -0.028 | -0.100 | 0.135 |
| GDF-15 | **-0.358** | 1 | **0.460** | 0.011 | **-0.424** | **-0.418** | **-0.469** | 0.073 | 0.009 | -0.184 |
| Follistatin | **-0.502** | **0.460** | 1 | -0.047 | **-0.499** | **-0.405** | **-0.590** | 0.147 | 0.058 | -0.163 |
| FSTL3 | 0.203 | 0.011 | -0.047 | 1 | 0.124 | 0.029 | 0.162 | 0.028 | -0.012 | -0.038 |
| LH | **0.354** | **-0.424** | **-0.499** | 0.124 | 1 | **0.643** | **0.635** | -0.145 | -0.079 | 0.034 |
| FSH | **0.310** | **-0.418** | **-0.405** | 0.029 | **0.643** | 1 | **0.357** | -0.279 | -0.161 | -0.060 |
| Estradiol | **0.473** | **-0.469** | **-0.590** | 0.162 | **0.635** | **0.357** | 1 | -0.198 | -0.242 | -0.029 |
| Leptin | -0.028 | 0.073 | 0.147 | 0.028 | -0.145 | -0.279 | -0.198 | 1 | **0.646** | **0.582** |
| BMI | -0.100 | 0.009 | 0.058 | -0.012 | -0.079 | -0.161 | -0.242 | **0.646** | 1 | **0.871** |
| BMI-SDS | 0.135 | -0.184 | -0.163 | -0.038 | 0.034 | -0.060 | -0.029 | **0.582** | **0.871** | 1 |

Values from HC group (both HC_OCP+ and HC_OCP-): were used (n=120 total; for Leptin n=110; for LH, FSH, Estradiol n=111). Bold font indicates significant correlations after adjusting the p-value for the number of multiple comparisons which were 28 (i.e. p<1.8x10^-3^).

**Supplemental Table 8:** HC group (both HC_OCP+ and HC_OCP-): Exact p-values of correlation coefficients based on spearman correlation analyses of the plasma concentrations of activin A, follistatin, GDF-15, and FSTL3 with the plasma concentrations of sex hormones and with BMI.

| P-values | Activin A | GDF-15 | Follistatin | FSTL3 | LH | FSH | Estradiol | Leptin | BMI | BMI-SDS |
| --- | --- | --- | --- | --- | --- | --- | --- | --- | --- | --- |
| Activin A |  | **6x10^-5^** | **5x10^-9^** | 0.026 | **1x10^-4^** | **9x10^-4^** | **2x10^-7^** | 0.774 | 0.275 | 0.142 |
| GDF-15 | **6x10^-5^** |  | **1x10^-7^** | 0.905 | **3x10^-6^** | **5x10^-6^** | **2x10^-7^** | 0.449 | 0.924 | 0.044 |
| Follistatin | **5x10^-9^** | **1x10^-7^** |  | 0.609 | **2x10^-8^** | **1x10^-5^** | **1x10^-11^** | 0.126 | 0.531 | 0.075 |
| FSTL3 | 0.026 | 0.905 | 0.609 |  | 0.196 | 0.762 | 0.089 | 0.771 | 0.897 | 0.678 |
| LH | **1x10^-4^** | **3x10^-6^** | **2x10^-8^** | 0.196 |  | **3x10^-14^** | **7x10^-14^** | 0.148 | 0.407 | 0.726 |
| FSH | **9x10^-4^** | **5x10^-6^** | **1x10^-5^** | 0.762 | **3x10^-14^** |  | **1x10^-4^** | 0.005 | 0.091 | 0.529 |
| Estradiol | **2x10^-7^** | **2x10^-7^** | **1x10^-11^** | 0.089 | **7x10^-14^** | **1x10^-4^** |  | 0.047 | 0.010 | 0.765 |
| Leptin | 0.774 | 0.449 | 0.126 | 0.771 | 0.148 | 0.005 | 0.047 |  | **2x10^-14^** | **3x10^-11^** |
| BMI | 0.275 | 0.924 | 0.531 | 0.897 | 0.407 | 0.091 | 0.010 | **2x10^-14^** |  | **3x10^-38^** |
| BMI-SDS | 0.142 | 0.044 | 0.075 | 0.678 | 0.726 | 0.529 | 0.765 | **3x10^-11^** | **3x10^-38^** |  |

Bold font indicates significant correlations after adjusting the p-value for the number of multiple comparisons which were 28 (i.e. p<1.8x10^-3^).

**Supplemental Table 9:** recAN group (both recAN_OCP+ and recAN_OCP-): Correlation coefficients based on spearman correlation analyses of the plasma concentrations of activin A, GDF-15, follistatin and FSTL3 with the plasma concentrations of sex hormones, leptin and with BMI as well as BMI-SDS.

| Spearman r | Activin A | GDF-15 | Follistatin | FSTL3 | LH | FSH | Estradiol | Leptin | BMI | BMI-SDS |
| --- | --- | --- | --- | --- | --- | --- | --- | --- | --- | --- |
| Activin A | 1 | **-0.396** | **-0.651** | 0.227 | **0.530** | **0.522** | **0.597** | -0.013 | 0.104 | 0.092 |
| GDF-15 | **-0.396** | 1 | **0.505** | 0.137 | -0.228 | -0.300 | **-0.458** | 0.105 | -0.018 | -0.036 |
| Follistatin | **-0.651** | **0.505** | 1 | -0.018 | **-0.516** | **-0.535** | **-0.681** | 0.120 | -0.091 | -0.111 |
| FSTL3 | 0.227 | 0.137 | -0.018 | 1 | 0.198 | 0.057 | 0.104 | 0.254 | 0.119 | 0.087 |
| LH | **0.530** | -0.228 | **-0.516** | 0.198 | 1 | **0.750** | **0.594** | 0.018 | 0.148 | 0.115 |
| FSH | **0.522** | -0.300 | **-0.535** | 0.057 | **0.750** | 1 | **0.425** | -0.039 | 0.133 | 0.121 |
| Estradiol | **0.597** | **-0.458** | **-0.681** | 0.104 | **0.594** | **0.425** | 1 | -0.006 | 0.073 | 0.048 |
| Leptin | -0.013 | 0.105 | 0.120 | 0.254 | 0.018 | -0.039 | -0.006 | 1 | **0.439** | **0.464** |
| BMI | 0.104 | -0.018 | -0.091 | 0.119 | 0.148 | 0.133 | 0.073 | **0.439** | 1 | **0.961** |
| BMI-SDS | 0.092 | -0.036 | -0.111 | 0.087 | 0.115 | 0.121 | 0.048 | **0.464** | **0.961** | 1 |

Values from recAN group (both recAN_OCP+ and recAN-) were used (n=80 total; for Leptin n=74; for LH, FSH, Estradiol n=77). Bold font indicates significant correlations after adjusting the p-value for the number of multiple comparisons which were 28 (i.e. p<1.8x10^-3^).

**Supplemental Table 10:** recAN group (both recAN_OCP+ and recAN_OCP-): Exact p-values of correlation coefficients based on spearman correlation analyses of the plasma concentrations of activin A, follistatin, GDF-15, and FSTL3 with the plasma concentrations of sex hormones and with BMI.

| P-values | Activin A | GDF-15 | Follistatin | FSTL3 | LH | FSH | Estradiol | Leptin | BMI | BMI-SDS |
| --- | --- | --- | --- | --- | --- | --- | --- | --- | --- | --- |
| Activin A |  | **3x10^-4^** | **6x10^-11^** | 0.042 | **7x10^-7^** | **1x10^-6^** | **10x10^-8^** | 0.913 | 0.358 | 0.419 |
| GDF-15 | **3x10^-4^** |  | **2x10^-6^** | 0.227 | 0.046 | 0.008 | **3x10^-5^** | 0.375 | 0.875 | 0.749 |
| Follistatin | **6x10^-11^** | **2x10^-6^** |  | 0.871 | **2x10^-6^** | **5x10^-7^** | **1x10^-11^** | 0.310 | 0.422 | 0.329 |
| FSTL3 | 0.042 | 0.227 | 0.871 |  | 0.084 | 0.625 | 0.368 | 0.029 | 0.293 | 0.445 |
| LH | **7x10^-7^** | 0.046 | **2x10^-6^** | 0.084 |  | **4x10^-15^** | **1x10^-8^** | 0.883 | 0.199 | 0.321 |
| FSH | **1x10^-6^** | 0.008 | **5x10^-7^** | 0.625 | **4x10^-15^** |  | **1x10^-4^** | 0.747 | 0.248 | 0.296 |
| Estradiol | **10x10^-8^** | **3x10^-5^** | **1x10^-11^** | 0.368 | **1x10^-8^** | **1x10^-4^** |  | 0.958 | 0.528 | 0.676 |
| Leptin | 0.913 | 0.375 | 0.310 | 0.029 | 0.883 | 0.747 | 0.958 |  | **9x10^-5^** | **3x10^-5^** |
| BMI | 0.358 | 0.875 | 0.422 | 0.293 | 0.199 | 0.248 | 0.528 | **9x10^-5^** |  | **2x10^-45^** |
| BMI-SDS | 0.419 | 0.749 | 0.329 | 0.445 | 0.321 | 0.296 | 0.676 | **3x10^-5^** | **2x10^-45^** |  |

Bold font indicates significant correlations after adjusting the p-value for the number of multiple comparisons which were 28 (i.e. p<1.8x10^-3^).

**References**

1. Fichter MM, Quadflieg N. Strukturiertes Inventar für anorektische und bulimische Esstörungen (SIAB); Fragebogen (SIAB-S) und Interview (SIAB-EX) nach DSM-IV und ICD-10; . Handanweisung Hogrefe, Göttingen, Germany. 1999.

2. Bargiacchi A, Clarke J, Paulsen A, Leger J. Refeeding in anorexia nervosa. Eur J Pediatr. 2019;178(3):413-22.
